# Sensitivity of intraoperative electrophysiological monitoring for scoliosis correction in identifying postoperative neurological deficits: a retrospective chart review of the Scoliosis Research Society morbidity and mortality database

**DOI:** 10.1186/s12891-024-08115-4

**Published:** 2025-02-24

**Authors:** Kenney Ki Lee Lau, Kenny Yat Hong Kwan, Jason Pui Yin Cheung

**Affiliations:** Department of Orthopaedics and Traumatology, School of Clinical Medicine, Li Ka Shing Faculty of Medicine, The University of Hong Kong, Pokfulam, Hong Kong

**Keywords:** Scoliosis, Intraoperative electrophysiological monitoring, Neurological deficits, Sensitivity analysis, Electromyography, Neurogenic motor evoked potential, Somatosensory evoked potential, Transcranial motor evoked potential

## Abstract

**Background:**

Surgical intervention is the ultimate treatment for scoliosis, but iatrogenic spinal cord injury is one of the major concerns. Although intraoperative electrophysiological monitoring can aid in detecting and reducing postoperative neurological complications, its use is still controversial.

**Methods:**

A retrospective chart review of 6,577 scoliotic patients who underwent surgery for curve correction with a reported complication was conducted. Our dataset was sourced from the morbidity and mortality database of the Scoliosis Research Society spanning the period from 2013 to 2023. The sensitivity of intraoperative monitoring was evaluated.

**Results:**

Intraoperative monitoring was used in 60% of surgeries, while 26% of the reported complications in the study cohort were new postoperative neurologic deficits. The overall monitoring performance indicated a sensitivity of 45%. Neurogenic motor evoked potential showed the best outcomes among the individual monitoring methods. The highest sensitivity (60.4%) was achieved using four monitoring methods, demonstrating significantly better results than one, two, and three methods.

**Conclusions:**

The monitoring practice benefits in distinguishing postoperative neurologic deficits within the scoliosis population. Employing four monitoring techniques yielded the most favourable outcomes.

## Introduction

Scoliosis, a complex three-dimensional deformity of the spine, affects millions of people worldwide and can lead to significant physical and psychological morbidity [1]. Surgical interventions continue to serve as the sole evidence-based treatment option with a notable success rate for correcting the curvature, aiming to hinder curve progression, improve spinal alignment, and alleviate associated pain and functional impairments [2]. However, one of the major concerns during the operation is the risk of iatrogenic spinal cord injury due to intraoperative cord compression, ischaemia, traction or direct mechanical damage [3]. Its subsequent neurologic deficits can be transient or permanent, resulting in sensorimotor disturbance or paraplegia [4]. Hence, the early detection and prevention of these complications are crucial to achieving optimal surgical outcomes.

Historically, the wake-up test, an intraoperative assessment, has presented challenges for anaesthesiologists and risks due to patient movement while only providing a single timepoint evaluation [5]. Intraoperative electrophysiological monitoring is a reliable indicator of impending spinal cord injury and aids in reducing the risk of postoperative neurological complications [6–10]. It involves continuously assessing the integrity of the spinal cord by measuring the electrical signals generated by the nervous system in response to various stimuli [11]. Its cost-effectiveness, compared with the lifetime costs associated with paraplegia, has led to the adoption of the monitoring technique in many hospitals [12]. Nevertheless, the monitoring in scoliosis surgery has yet to be fully implemented. Its absence in some countries could be attributed to resource and training insufficiencies [13].

As such, the primary aim of this study was to reinforce the significance of intraoperative electrophysiological monitoring during scoliosis correction procedures. Utilising data obtained from a large-scale multicentred database, we intended to furnish robust evidence supporting the clinical relevance of the monitoring. Our objective was to assess the sensitivity of the monitoring techniques in patients with scoliosis and to identify the most effective approach or combination of approaches that would yield the highest sensitivity. This information would prompt healthcare providers to reevaluate the integration of the monitoring. In doing so, we endeavoured to contribute to improving safety in surgical procedures for spinal deformities.

## Methods

The study design of this research is a retrospective chart review. Ethics approval is exempt because of the use of anonymised data [14].

### Source

The Scoliosis Research Society is an international organisation committed to improving patient care for spinal deformities. Since its establishment in 1965, it has collected morbidity and mortality data, making its database one of the largest global registries. The value of this database comes from the substantial number of patient records it contains and its multi-institutional nature. The database is populated with procedures reported by surgeons who have completed their fellowship training and are either active or candidate members of the society. Candidate members must provide all spinal case data over five years to achieve active membership, after which they must report annually or choose to pay an exception fee. Submissions were made via an online survey, with members being prompted to document operative, perioperative, and postoperative complications within 30 days following surgery. The data extraction process spanned between the years 2013 and 2023.

### Procedures

A proposal for morbidity and mortality research was developed for review by the outcomes and benchmarking committee of the society. Subsequent to its approval, the data extraction was carried out by the database administrator. The funding for this data retrieval was provided by the society as a benefit extended to its members.

### Eligibility

Patients with scoliosis who have undergone surgical intervention with a reported complication were incorporated into this study. The exclusion criteria consisted of individuals with post-traumatic scoliosis and those missing data on intraoperative monitoring and newly developed postoperative neurologic deficits.

### Variables

Of the elements documented in the database, we extracted patient demographics such as age, sex, American Society of Anesthesiologists (ASA) physical status classification, and past medical history (i.e., smoking, diabetes, hypertension, vascular issues, cardiac problems, pulmonary issues, thromboembolic events, prior spine infections, and cancer). Additionally, data on the type of scoliosis, magnitude of the major curve, surgical aspects (i.e., operative time, direct neural decompression usage, fusion application, fusion category, number of segments fused, osteotomy employment, and implant utilisation), monitoring details (i.e., use of electrophysiological monitoring, type of monitoring technique, and detected abnormalities), and complication information (i.e., presence of new postoperative neurologic deficits, onset of the deficits, region of the spinal cord injuries, cord level of the injuries, and recovery of the deficits) were also obtained.

### Measures

True positive cases reflected abnormalities identified through intraoperative monitoring, with patients subsequently experiencing new postoperative neurological deficits. Conversely, false negative instances indicated that abnormalities were not recognised during the surgical procedure, yet patients still developed the deficits. Sensitivity describes the percentage of patients exhibiting postoperative neurological impairments who were accurately identified through monitoring [15].

### Statistics

The original raw data was processed using MATLAB packages (version 2024a, MathWorks, United States). In light of the study objective, sensitivity was employed to evaluate a range of intraoperative monitoring techniques. Subgroup analysis was performed using the chi-square test. Statistical analyses were conducted using SPSS software (version 29.0, International Business Machines, United States), and a significance level of 0.05 was established for the analyses.

## Results

Initially, we identified 6,647 records of scoliosis surgeries with complications in the database. Following the removal of cases due to post-traumatic scoliosis (*n* = 26), lack of monitoring data (*n* = 42), and absence of new postoperative neurological deficit data (*n* = 7), the final samples for the present study comprised 6,577 patient reports.

The monitoring technique was utilised in 59.8% of the surgical procedures. Subsequently, 25.8% of the reported complications in the study cohort were new postoperative neurological deficits. Table 1 delineates the characteristics of all participating patients, and Table 2 displays the features of the surgeries for scoliosis correction. Table 3 encompasses the details of intraoperative monitoring, and Table 4 presents an overview of the postoperative deficits.

**Table 1:** Characteristics of patients

Factor	Mean ± standard deviation / Proportion
**Demographics**	
Age	30 ± 25 years
Sex	64.6% females
Physical Status	ASA I: 28.3% ASA II: 30.2% ASA III: 35.0% ASA IV: 6.4% ASA V: 0.1%
**History**	
Smoking	3.4%
Diabetes	7.6%
Hypertension	18.8%
Vascular diseases	4.9%
Cardiac diseases	9.7%
Pulmonary diseases	17.0%
Thromboembolic diseases	1.9%
Previous spine infection	5.7%
Cancer	2.6%
**Scoliosis**	
Type	Idiopathic: 30.8% Degenerative: 21.9% Neuromuscular: 28.0% Congenital: 10.1% Syndromic: 9.1%
Magnitude	≤ 30°: 8.4% 31°–40°: 8.8% 41°–50°: 11.3% 51°–60°: 17.1% 61°–70°: 16.3% 71°–80°: 12.8% 81°–90°: 10.8% 91°–100°: 6.9% ≥ 100°: 7.6%

**Table 2:** Characteristics of surgeries

Factor	Mean ± standard deviation / Proportion
Operative time	< 2 h: 6.5% 2–6 h: 61.1% 6–9 h: 25.6% 9–12 h: 5.4% > 12 h: 1.4%
Direct neural decompression	28.2%
Fusion performed	80.9%
Fusion category	Anterior: 2.1% Anterior and Posterior: 15.8% Posterior or Posterolateral: 80.8% Limited fusion at anchor site: 1.3%
Number of segments fused	12 ± 5 levels
Osteotomy performed	53.3%
Implants used	89.3%

**Table 3:** Details of the intraoperative electrophysiological monitoring

Factor	Proportion
Use of electrophysiological monitoring	59.8%
Types of method	
Electromyography	65.0%
Somatosensory evoked potential	87.2%
Neurogenic motor evoked potential	31.6%
Transcranial motor evoked potential	61.0%
Number of methods	
Monitoring technique used	One monitor: 14.1% Two monitors: 34.2% Three monitors: 44.6% Four monitors: 7.1%
Indication of neurological problems	
Abnormalities detected	20.0%
Prolonged latency	8.4%
Decreased amplitude	18.4%
Positive electromyography potentials	1.8%

**Table 4:** Particulars of the postoperative neurologic deficits

Factor	Proportion
New postoperative neurologic deficits	25.8%
Onset of the deficits	Intraoperative: 32.1% Acute postoperative (within 12 h): 32.7% Acute postoperative (12–24 h): 11.7% Delayed postoperative (after 24 h): 23.5%
Region of the deficits	Cervical: 4.9% Thoracic: 41.1% Lumbar: 52.3% Sacrum: 1.7%
Cord level of the deficits	77.6% incomplete
Recovery of the deficits	Complete: 53.1% Partial: 40.6% None: 6.3%

In consideration of our primary objective, we offered a set of diagnostic indicators for the monitoring of the deficits through the utilisation of diverse methods and their combinations. Overall, the performance of the monitoring had 638 true positives and 774 false negatives, with a sensitivity of 45.2%. Among the various monitoring methods, neurogenic motor evoked potential exhibited the best sensitivity (51.2%). Electromyography had a sensitivity of 43.0%, whereas somatosensory evoked potential showed a sensitivity of 46.1%. Meanwhile, transcranial motor evoked potential displayed a sensitivity of 49.4%. Utilising a single monitoring method resulted in a sensitivity of 39.8%. When two monitoring methods were employed, the sensitivity slightly increased to 41.9%. With the use of three monitoring methods, the sensitivity improved to 46.9%. The optimal performance was achieved when four monitoring methods were applied, reaching a sensitivity of 60.4%. The information on the outcomes of the monitoring is delineated in Table 5.

**Table 5:** Diagnostic indicators for the monitoring of neurologic deficits

Monitoring	True positive	False negative	Sensitivity
Overall	638	774	45.2%
Electromyography	403	534	43.0%
Somatosensory evoked potential	559	654	46.1%
Neurogenic motor evoked potential	231	220	51.2%
Transcranial motor evoked potential	422	433	49.4%
One monitoring method	80	121	39.8%
Two monitoring methods	203	281	41.9%
Three monitoring methods	291	330	46.9%
Four monitoring methods	64	42	60.4%

Notably, there were no significant differences observed between the use of one, two, and three monitoring methods (i.e., one method compared to two methods: *p* = 0.604; one method compared to three methods: *p* = 0.080; two methods compared to three methods: *p* = 0.103). However, the implementation of four monitoring methods yielded significantly superior results in comparison to one (*p* < 0.001), two (*p* < 0.001), and three monitoring methods (*p* = 0.010).

## Discussion

Our study has retrieved 6,577 scoliotic patient data with reported complications from the morbidity and mortality database of the Scoliosis Research Society for further analysis. Intraoperative electrophysiological monitoring was utilised in 59.8% of the cases, and new postoperative neurologic deficits encompassed the complications reported in 25.8% of patients. We found that the overall intraoperative monitoring for postoperative neurological complications has a sensitivity of 45.2%.

Some systematic reviews with meta-analyses have illustrated the diagnostic accuracy of different monitoring techniques. For solely somatosensory evoked potential, the pooled sensitivity amounted to 84% (95% confidence intervals: 59–95%).^6^ In the case of combined somatosensory evoked potential and transcranial motor evoked potential, the pooled sensitivity was 82.6% (95% confidence intervals: 57–95%).^7^ As for transcranial motor evoked potential exclusively, the pooled sensitivity was 91% (95% confidence intervals: 34–100%).^8^ Regrettably, neurogenic motor evoked potential has not been the subject of any reviews. Although the current results exhibited relatively lower sensitivity than the existing literature, it is essential to note that our study encompassed all types of scoliosis cases. In contrast, those reviews specifically addressed adolescent idiopathic scoliosis.

Each method of intraoperative electrophysiological monitoring comes with its advantages and drawbacks. Somatosensory evoked potential is often utilised due to its non-invasive characteristics and capacity to offer feedback about the functional integrity of sensory pathways [16]. However, its sensitivity is primarily focused on dorsal column function and may not accurately reflect the integrity of motor pathways. Motor evoked potential has demonstrated significant accuracy in identifying abnormalities as it directly monitors motor pathways [17]. Nevertheless, it can be affected by factors such as anaesthetic agents and patient temperature, which can impact its reliability. Electromyography can identify nerve irritation or damage by tracking the electrical activity of muscles, but it serves more as a broad monitor and is not capable of predicting minor alterations in neurological function [18].

Our findings affirmed the effectiveness of the monitoring as a tool for identifying neurological issues during and after surgery. With a sensitivity rate that could reach over 60%, we provided compelling evidence for the value of the monitoring. Although neurogenic motor evoked potential revealed the highest sensitivity, the utilisation of four monitoring methods produced the most favourable outcomes. Since multimodality monitoring increases the sensitivity of detecting abnormalities by providing multiple signals [19], it might reduce the risk of relying on a single method that does not fully capture all potential neurological problems [20, 21]. Employing a combination of monitoring techniques can mitigate some of their individual shortcomings, thereby offering a more thorough assessment of both sensory and motor pathway functionality. Nonetheless, this strategy necessitates additional resources [22], such as specialised personnel for accurate interpretation and may increase the complexity of the procedure.

While our study offered important insights into intraoperative monitoring in scoliosis surgery, there are still knowledge gaps that future research could address. There is currently no consensus on the optimal combination of monitoring techniques for scoliosis surgery. Although intraoperative monitoring has been linked to improved surgical outcomes, its cost-effectiveness remains uncertain. Furthermore, the impact of the proficiency level and standardisation of monitoring procedures on detecting neurological abnormalities is still unclear. The effect of different types and levels of anaesthesia on the efficacy of intraoperative monitoring is not fully understood.

Several approaches could be implemented to encourage the utilisation of intraoperative monitoring. Offering specialised training in the application and interpretation of the monitoring may contribute to the better performance of the monitoring process. Allocating resources to research and development to enhance the reliability and accuracy of the monitoring may further improve their effectiveness in detecting and managing neurological complications during and after surgery. Undertaking cost-effectiveness analyses would assist healthcare providers and policymakers in comprehending the long-term benefits of the monitoring, such as decreased healthcare expenses.

Limitations in the present study warrant acknowledgement. First, our study employed a retrospective design, which may introduce potential biases and inaccuracies in the data. Second, we did not sufficiently account for possible confounding factors that could impact the measures, like the surgeon’s level of experience. Third, we primarily concentrated on short-term postoperative outcomes without comprehensively analysing long-term neurological recovery. Lastly, our study lacks in-depth information regarding the monitoring technique itself, the expertise of the monitoring team, and the management of abnormalities detected during surgery.

## Conclusions

This study analysed the database from the Scoliosis Research Society and demonstrated the benefits of intraoperative electrophysiological monitoring in detecting postoperative neurologic deficits within the scoliosis population. With an overall sensitivity of 45.2%, using four monitoring techniques yielded the most favourable outcomes (i.e., 60.4%).

## Data Availability

The datasets used and/or analysed during the current study are available from the corresponding author upon reasonable request.
